# Depression and type 2 diabetes risk: a Mendelian randomization study

**DOI:** 10.3389/fendo.2024.1436411

**Published:** 2024-08-29

**Authors:** Kaiyuan Liu, Diyi Zhou, Lijun Chen, Sida Hao

**Affiliations:** ^1^ Department of Endocrinology, Zhejiang Integrated Traditional Chinese and Western Medicine Hospital, Hangzhou, Zhejiang, China; ^2^ Department of Urology, Zhejiang Integrated Traditional Chinese and Western Medicine Hospital, Hangzhou, Zhejiang, China

**Keywords:** major depression, depressive status, type 2 diabetes, Mendelian randomization, casual relationship

## Abstract

**Background:**

Extensive observational evidence has suggested an association between depression and type 2 diabetes (T2D). However, the causal relationships between these two diseases require further investigation. This study aimed to evaluate the bidirectional causal effect between two types of depression and T2D using two-sample Mendelian randomization (MR).

**Methods:**

We applied two-step MR techniques, using single-nucleotide polymorphisms (SNPs) as the genetic instruments for analysis. We utilized summary data from genome-wide association studies (GWASs) for major depression (MD), depressive status (frequency of depressed mood in the last two weeks), T2D, and other known T2D risk factors such as obesity, sedentary behavior (time spent watching television), and blood pressure. The analysis utilized inverse variance weighted (IVW), MR-Egger regression, weighted median, weighted mode, MR pleiotropy residual sum, and outlier methods to determine potential causal relationships.

**Results:**

The study found that MD was positively associated with T2D, with an odds ratio (OR) of 1.26 (95% CI: 1.10-1.43, p = 5.6×10^-4^) using the IVW method and an OR of 1.21 (95% CI: 1.04-1.41, p = 0.01) using the weighted median method. Depressive status was also positively associated with T2D, with an OR of 2.26 (95% CI: 1.03-4.94, p = 0.04) and an OR of 3.62 (95% CI: 1.33-9.90, p = 0.01) using the IVW and weighted median methods, respectively. No causal effects of MD and depressive status on T2D risk factors were observed, and T2D did not influence these factors.

**Conclusion:**

Our study demonstrates a causal relationship between depression and an increased risk of developing T2D, with both major depression and depressive status being positively associated with T2D.

## Introduction

The incidence of type 2 diabetes (T2D) has risen sharply worldwide in recent years, driven by changes in environmental factors, behavior, and lifestyle (1). In 2021, the global prevalence of diabetes among adults aged 20-79 was estimated at 536.6 million, representing over 10% of the world’s adult population, with projections suggesting this number will rise to 783 million by 2045 (2). T2D, the most common form of diabetes, results from a complex interplay between genetic and environmental factors (3). Key environmental and psychological factors linked to T2D include rapid economic development, urbanization, sedentary lifestyles, unhealthy diets, and depression (4).

Depression, a prevalent psychological condition, has been identified as a significant risk factor for T2D. The World Health Organization estimates that depression will become the leading cause of disability worldwide by 2030 (5). Studies have shown a significant correlation between T2D and depression. A nationally representative cross-sectional study indicated that T2D patients have the highest incidence of depression at 17.15%, particularly among those with poor blood glucose control (6). Additionally, a retrospective cohort study in the UK and USA found an increased risk of depression among T2D patients, especially younger individuals, irrespective of other comorbidities (7). However, another study suggested a reverse causal relationship, where depression increased the incidence of T2D by 52%, with a synergistic effect with obesity (8). A meta-analysis of cohort studies also showed that there was a moderate bidirectional correlation between depression and T2D, but there is no direct causal evidence (9).

The causality between T2D and depression remains contentious. Mendelian randomization (MR) offers a robust method to infer causality, using genetic variants as instrumental variables. Genome-wide association studies (GWASs) were conducted in European and Asian populations, and more than 600 new loci regulating T2D risk were identified (10). Previous MR studies have explored this relationship, but findings have been inconsistent, particularly after adjusting for confounders such as body mass index (BMI) (11). With the emergence of new genetic databases and advanced GWASs, our study seeks to re-examine the causal relationship between T2D and depression using updated data and refined methodologies. The purpose of this study was to investigate the bidirectional causal relationship between depression and T2D using two-sample MR. We hypothesized that both major depression and depressive status would have a causal impact on the risk of developing T2D, and conversely, that T2D would influence the risk of depression.

## Methods

### Overall study design

We used a two-step two-sample MR with publicly available datasets that provide genome-wide association results for major depression (MD), depressive status, T2D, and other known T2D risk factors, such as obesity, sedentary behavior (time spent watching television), and blood pressure. First, we used two sets of data to test the causal effects of MD and depressive status on T2D, and then the causal effects on T2D risk factors. Finally, we identified the causal effects of T2D on depression.

### Data sources

The genetic associations were estimated using data from several meta-analyses, the FinnGen consortium (https://www.finngen.fi/en), and the UK Biobank (UKBB) (https://www.ukbiobank.ac.uk). The details can be found in **Table 1**. Genetic variants for MD were obtained from the largest GWAS meta-analysis which included 170,756 cases and 329,443 control cases (12). To avoid confounding from the variation of depression severity, we constructed another set of genetic instruments based on the depressive status of a recent GWAS from the Medical Research Council-Integrative Epidemiology Unit (MRC-IEU) consortium which included 442,840 cases who experienced a particular frequency of depressed mood over 2 weeks. The GWAS summary data for T2D were obtained from a meta-analysis (13) and the FinnGen consortium. There were 62,892 T2D cases and 596,424 control cases in the meta-analysis and 32,469 T2D cases and 183,185 control cases in the FinnGen study. The GWAS summary data for T2D risk factors, including obesity, time spent watching television, systolic blood pressure, and diastolic blood pressure, were extracted from the FinnGen study, the UK Biobank, and the International Consortium of Blood Pressure (14) separately. Individuals who had withdrawn consent were excluded from all data sources. All cases and control cases in these studies had European ancestry and there was no significant overlap between GWAS populations.

**Table 1:** Details of studies included in the Mendelian randomization analyses.

Phenotype	Participants	PubMed ID or URL
Major depression	500,199	30718901
Frequency of depressed mood in last 2 weeks	442,840	MRC-IEU consortium
Type 2 diabetes	215,654	FinnGen consortium
	655,666	30054458
Obesity	342,400	FinnGen consortium
Time spent watching television	437,887	UK Biobank
Systolic blood pressure	757,601	30224653
Diastolic blood pressure	757,601	30224653

### Instrumental variable selection

In this study, single nucleotide polymorphisms (SNPs) were chosen as the instrumental variables (IVs) to investigate the causal relationships between depression (MD and depressive status) and T2D. The MD and depressive status served as the exposure, whereas T2D served as the outcome. The SNPs should satisfy three assumptions: Relevance—the SNPs should have a strong association with the exposure variable; Independence—the SNPs are unrelated to any potential confounders; Exclusion—the impact of the SNPs on the outcome is solely through the exposure variable and does not affect the outcome via any other pathways (15). First, the SNPs that were significantly related to the exposure were selected as the IVs. The selected SNPs that served as IVs had a threshold less than the genome-wide statistical significance threshold (5×10^−8^). Second, there was no linkage disequilibrium (LD) among the SNPs (*R* ^2^ < 0.01 and clumping distance=10,000 kb). Third, the SNPs that were related to any potential confounders were removed to satisfy the independence assumption. Fourth, to ensure that the effects of the SNPs on exposure corresponded to the same allele as the effects on the outcome, palindromic SNPs with ambiguous allele frequencies were removed.

### Statistical analyses

In this study, multiple methods including inverse variance weighted (IVW) (16), MR-Egger regression (17), weighted median (18), weighted mode (19), and MR pleiotropy residual sum and outlier (MR-PRESSO) (20) were used to examine whether there was a causal association between depression and T2D. The IVW method is reported to be more powerful than the others under certain conditions, so we used the IVW method as the main statistical model (16, 18), and the other methods were used to complement the IVW results. The weighted median method can generate consistent causal estimates assuming that at least 50% of the SNPs are valid. The MR-Egger regression can detect possible violations of instrumental variable assumptions due to directional horizontal pleiotropy, and a p-value of the intercept > 0.05 indicates no horizontal pleiotropic effects. Cochran’s *Q* test from the IVW approach tests the heterogeneity (21). In addition, a “leave-one-out” analysis was performed by sequentially omitting each instrumental SNP to discover potential heterogeneous SNPs. After removing the corresponding outliers, the MR-PRESSO method can detect outliers and provide a causal estimate from IVW. F-statistics were calculated to estimate the strength of the IVs and an F-statistic >10 suggested a sufficiently strong instrument (22). The results are presented as odds ratios (OR) and 95% confidence intervals (CI). All analyses were performed using the TwoSampleMR (23), MendelianRandomization (24), and MR-PRESSO packages (20) in R software (version 4.2.3).

## Results

### The causal effect of MD on T2D

**Figure 1:**
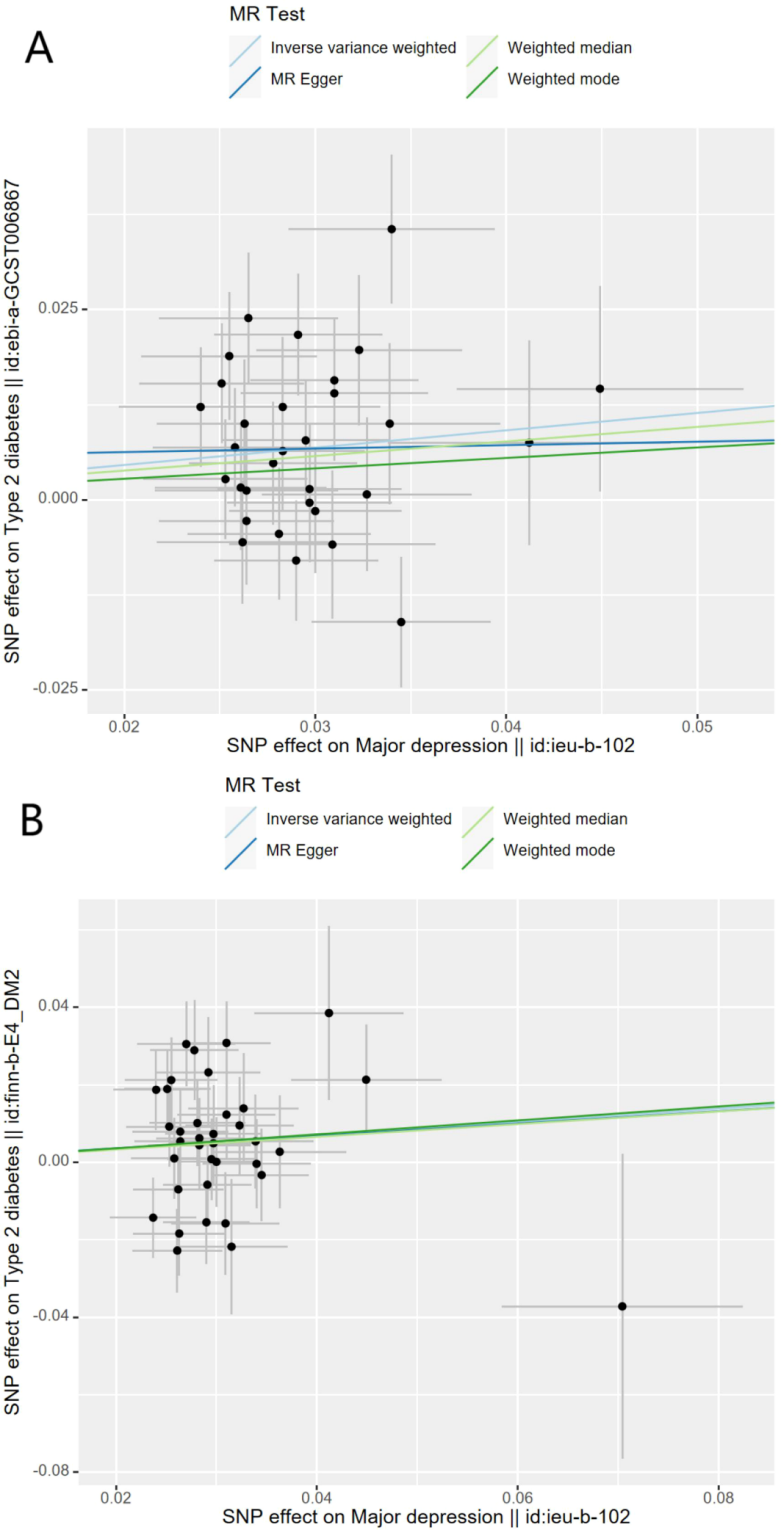
Scatter plots for Mendelian randomization analysis of major depression and the risk of type 2 diabetes. **(A)** Associations in the meta-analysis, **(B)** associations in FinnGen. Horizontal axis: SNPs’ association with major depression. Vertical axis: SNPs’ association with type 2 diabetes. The gradient of each line represents the MR estimate for the corresponding model.

**Figure 2:**
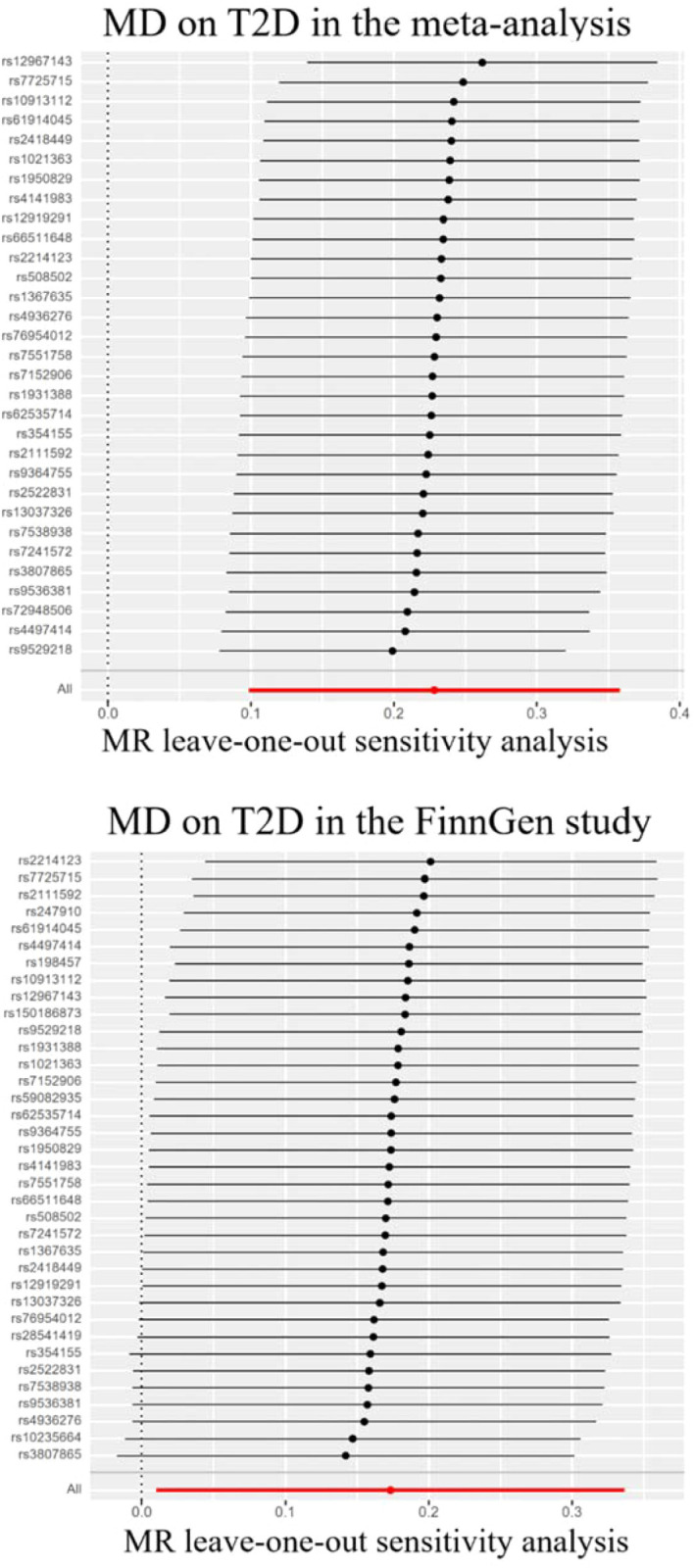
Leave-one-out sensitivity tests. We calculated the MR results of the remaining IVs after removing the IVs one by one. MD, major depression; T2D, type 2 diabetes.

Genetically predicted MD was positively associated with T2D in the meta-analysis and the FinnGen study. According to the IV selection criteria, 31 SNPs were used as IVs in the meta-analysis and 36 SNPs were selected in the FinnGen study. **Figure 1** shows the scatter plots and **Figure 2** shows the leave-one-out sensitivity tests for MR analysis. These 31 SNPs explained 1.1% of the variability in MD and the F-statistics of the IVs ranged from 140.24 to 249.48, indicating that the instruments had a strong potential to predict MD. Our IVW results showed strong evidence of a potential causal effect of MD on T2D with statistical significance (OR=1.26, 95%CI=1.10-1.43, p=5.6×10^−4)^. Furthermore, similar risk estimates were gained using the weighted median method (OR=1.21, 95% CI=1.04-1.41, p=0.01), while the results were not statistically significant using the MR-Egger approach (OR=1.05, 95%CI=0.36-3.20, p=0.93) and weighted mode method (OR=1.15, 95% CI=0.82-1.61, p=0.43). However, heterogeneity was observed with a Cochran Q-test with a p-value of 0.03 for IVW. MR-PRESSO also presented a similar result (the p-value in the global heterogeneity test=0.04). However, there was no significant directional horizontal pleiotropy according to the results of the MR-Egger regression intercept analysis (intercept=0.005; SE=0.01. p=0.74). In the FinnGen study, we found weak evidence of a potential causal effect of MD on T2D at statistical significance (OR=1.19, 95% CI=1.01-1.40, p=0.037). These 36 SNPs explained 1.2% of the variability in MD and the F-statistics of the IVs ranged from 137.79 to 249.48. The results were not statistically significant using the MR-Egger approach (OR=1.17, 95%CI=0.39-3.54, p=0.79), the weighted median method (OR=1.18, 95% CI=0.97-1.43, p=0.10), or the weighted mode method (OR=1.97, 95% CI=0.83-1.73, p=0.35). Meanwhile, heterogeneity was observed with a Cochran Q-test derived p-value of 0.02 for IVW. MR-PRESSO also presented a similar result (p-value in the global heterogeneity test=0.02). However, there was no evidence of a significant intercept (intercept=0.0004; SE=0.02; p=0.98), indicating that there was no observed directional pleiotropy. Among the two T2D databases, the IVW method showed that MD significantly increased the risk for T2D, and no pleiotropy was observed. Although some other methods had no statistical significance and heterogeneity existed, MD was positively associated with T2D (**Table 2**).

**Table 2:** Association of genetically predicted major depression with type 2 diabetes in sensitivity analyses.

Method	Value	*P*
Outcome Source: GWAS meta-analysis		
IVW method	OR, 1.26, (95% CI, 1.10-1.43)	5.6×10^−4^
MR-Egger regression	OR, 1.05, (95% CI, 0.36-3.02)	0.93
Weighted median method	OR, 1.21, (95% CI, 1.04-1.41)	0.01
Weighted mode method	OR, 1.15, (95% CI, 0.82-1.61)	0.43
Intercept in MR-Egger regression	—	0.74
Cochran’s Q test	46	0.03
Outcome Source: FinnGen		
IVW method	OR, 1.19, (95% CI, 1.01-1.43)	0.04
MR-Egger regression	OR, 1.17, (95% CI, 0.39-3.54)	0.79
Weighted median method	OR, 1.18, (95% CI, 0.97-1.43)	0.10
Weighted mode method	OR, 1.97, (95% CI, 0.83-1.73)	0.35
Intercept in MR-Egger regression	—	0.98
Cochran’s Q test	55	0.02

### The causal effect of depressive status on T2D

**Figure 3:**
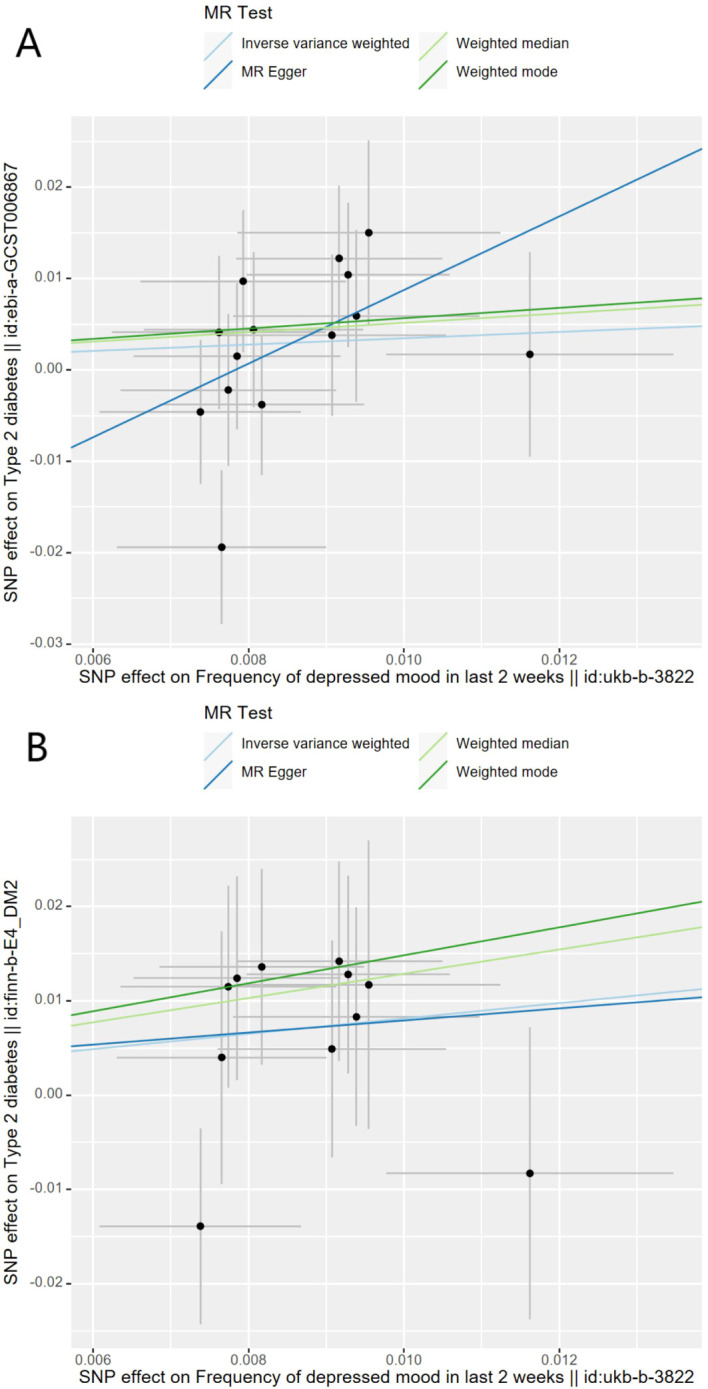
Scatter plots for Mendelian randomization analysis of depressive status and risk of type 2 diabetes. **(A)** Associations in the meta-analysis, **(B)** associations in FinnGen. Horizontal axis: SNPs’ association with frequency of depressed mood in last 2 weeks. Vertical axis: SNPs’ association with type 2 diabetes. The gradient of each line represents the MR estimate for the corresponding model.

**Figure 4:**
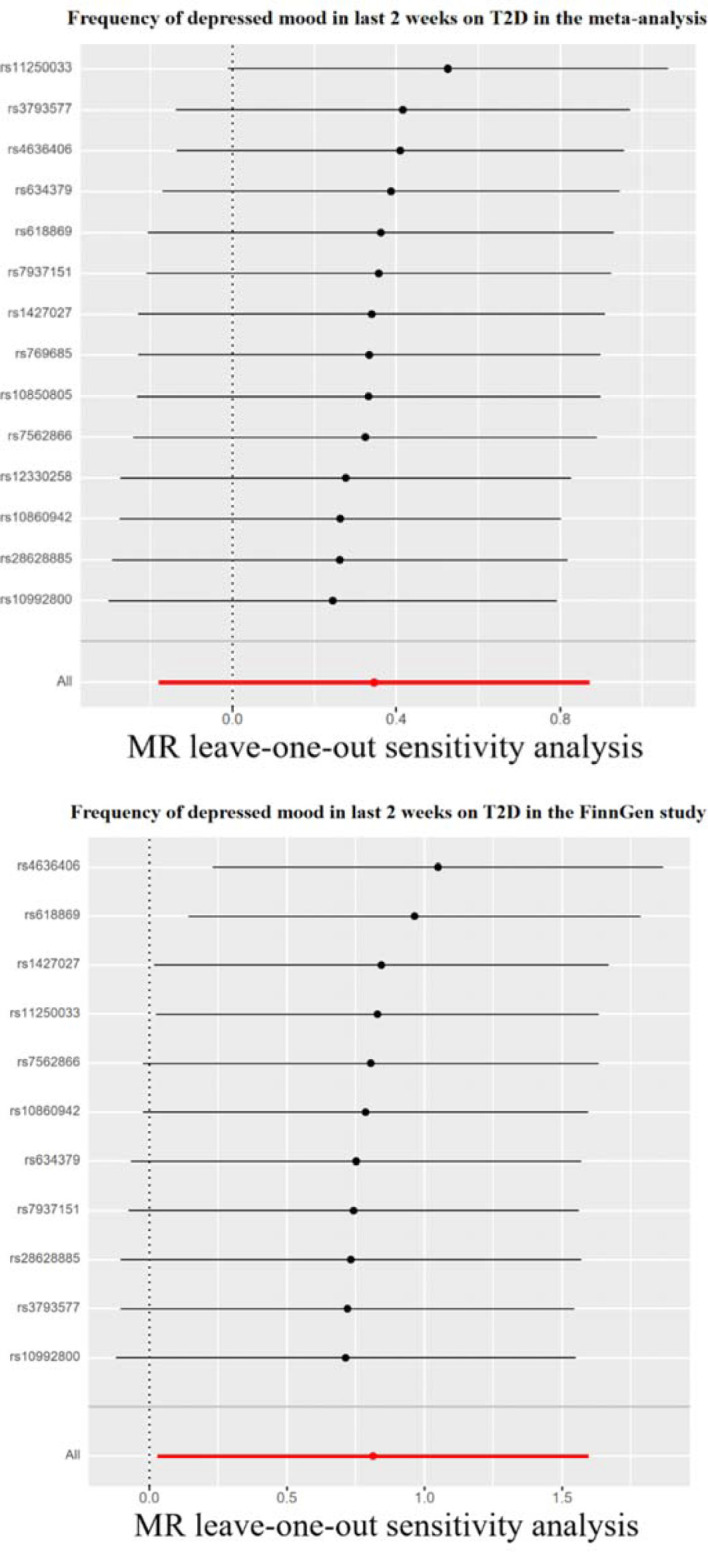
Leave-one-out sensitivity tests. We calculated the MR results of the remaining IVs after removing the IVs one by one. T2D, type 2 diabetes.

Genetically predicted depressive status was positively associated with T2D in the FinnGen study but had no causality on T2D in the meta-analysis. In the FinnGen study, 11 SNPs were significantly and independently associated with depressive status and explained 0.03% of the variability, and the F-statistics were all >10. **Figure 3** shows the scatter plots and **Figure 4** shows the leave-one-out of sensitivity tests for the MR analysis. The result of IVW indicated that depressive status was positively associated with T2D (OR=2.26, 95% CI =1.03-4.94, p=0.04) and there was no horizontal pleiotropy (p=0.96) or heterogeneity (p=0.64). MR-PRESSO also presented a similar result (p-value in the global heterogeneity test=0.78). Similar risk estimates were gained using the weighted median method (OR=3.62, 95% CI=1.33-9.90, p=0.01), while the results were not statistically significant using the MR-Egger approach (OR=1.90, p=0.85) or the weighted mode method (OR=4.41, p=0.08). In the meta-analysis, 14 SNPs were significantly associated with depressive status. The IVW result indicated that depressive status had no causality on T2D (OR=1.41, 95% CI =0.83-2.39, p=0.19) (**Table 3**).

**Table 3:** Association of genetically predicted depressive status with type 2 diabetes in sensitivity analyses.

Method	Value	*P*
Outcome Source: FinnGen		
IVW method	OR, 2.26, (95% CI, 1.03-4.94)	0.04
MR-Egger regression	OR, 1.90, (95% CI, 0.002-1469)	0.85
Weighted median method	OR, 3.62, (95% CI, 1.33-9.90)	0.01
Weighted mode method	OR, 4.41, (95% CI, 1.01-19.23)	0.08
Intercept in MR-Egger regression	—	0.96
Cochran’s Q test	6.8	0.75
Outcome Source: GWAS meta-analysis		
IVW method	OR, 1.41, (95% CI, 0.83-2.39)	0.20
MR-Egger regression	OR, 56.05, (95% CI, 0.58-5418)	0.11
Weighted median method	OR, 1.67, (95% CI, 0.82-3.39)	0.15
Weighted mode method	OR, 1.76, (95% CI, 0.58-5.37)	0.34
Intercept in MR-Egger regression	—	0.14
Cochran’s Q test	13	0.43

### The causal effect of depression on potential T2D risk factors

To determine whether the MR association between genetically determined depression and T2D was destroyed through the pleiotropic pathway associated with T2D, we used the IVW method to identify the relationship between depression and several T2D factors, such as obesity, time spent watching television, systolic blood pressure, and diastolic blood pressure. The databases of depressive status and time spent watching television were both from UKBB, which may lead to biased results, so we excluded this group. All p-values were larger than 0.05 and had no statistical significance. Thus, no causal effects of MD and depressive status on potential T2D risk factors were observed (**Tables 4**, **5**).

**Table 4:** Mendelian randomization estimation of the correlation between major depression and risk factors.

Outcome	Causal effect (95% CI)	*p-value*
Obesity	1.21(0.99-1.47)	0.06
Time spent watching television	1.01(0.98-1.04)	0.55
Systolic blood pressure	0.99 (0.57-1.73)	0.98
Diastolic blood pressure	10.75(0.52-1.07)	0.11

**Table 5:** Mendelian randomization estimation of the correlation between depressive status and risk factors.

Outcome	Causal effect (95% CI)	p-value
Obesity	1.95(0.93-4.09)	0.08
Systolic blood pressure	0.93(0.09-10.03)	0.95
Diastolic blood pressure	0.88(0.21-3.75)	0.86

### The causal effect of T2D on depression

We also performed a reverse MR. Using MD and depressive status as the outcomes and T2D as the exposure, we did not find a causal relationship based on IVW analysis. All p-values were larger than 0.05 and had no statistical significance. This showed that T2D did not affect MD and depressive status (**Table 6**).

**Table 6:** Mendelian randomization estimation of the correlation between Type 2 diabetes and depression.

Exposure	Outcome	Causal effect (95% CI)	*p-value*
Type 2 Diabetes: Finngen	Major depression	0.997 (0.98-1.01)	0.71
GWAS meta-analysis		0.999 (0.98-1.02)	0.83
Type 2 Diabetes: Finngen	Depressive status	1.00(0.998-1.01)	0.23
GWAS meta-analysis		1.01(1.00-1.01)	0.05

## Discussion

We applied a two-sample MR approach to comprehensively evaluate whether depression causally influences T2D incidence and whether T2D causally influences depression. We found evidence to support the causal role of genetically predicted MD and depressive status on the risk of T2D. There was no evidence that T2D increased the risk of depression incidence.

In a prospective analysis of population-based data from the UK Biobank (9,047 people with T2D and 68,739 people without diabetes) and the Maastricht Study (1,158 people with T2D and 3,372 people without diabetes), individuals with T2D had a higher risk of major depression (hazard ratio [HR] 1.61 [95% CI 1.49-1.77]) (25). In a cross-sectional study in China, during a 3-year observation, 316 patients with T2D were newly discovered from the 2,809 participants, and the relative risk and 95% confidence interval of depression on the incidence of diabetes was 1.52 (1.05-2.21). Depression was associated with a 52% increase in the incidence of type 2 diabetes (8). A study in Ontario, Canada, which included 59,315 adults living in communities, aimed to compare the incidence rate of T2D among adults with weight changes related to depression over the past 20 years. The results showed that compared with patients without depression, patients with depression without weight change or with weight gain had an increased risk of developing T2D (26). In a systematic review, there was moderate evidence that there was a causal relationship between T2D and the risk of depression, but more limited evidence showed that there was a causal relationship between depression and the risk of diabetes (27). Therefore, there is controversy over whether there is a causal relationship between depression and T2D, as well as which is the cause and which is the outcome. In addition, depression was associated with an excess mortality rate in patients with T2D recruited clinically in a Danish cohort of patients with T2D (n=8175) (28). A retrospective cohort study (29) in Italy found the same result, as depression was associated with increased T2D complications and mortality. We urgently need to clarify the causal relationship between depression and T2D. The discrepancies across these studies might be caused by residual confounders.

There are several underlying mechanisms that support that MD and depression could increase the incidence rate of T2D. Depression mostly occurs in early adulthood and is associated with self-neglect and inferiority, which may increase the risk of unhealthy lifestyles such as high BMI, poor diet, low levels of physical activity, and smoking, all of which in turn increase the risk of developing T2D (30). Studies (31, 32) showed that patients with depression and subthreshold depression often suffered from poorer self-care and poor lifestyle self-management such as diet, exercise, and managing elevated blood sugar levels. Patients with MD usually have an overactive hypothalamus pituitary adrenal (HPA) axis. An important function of the HPA axis is to stimulate cortisol secretion and promote gluconeogenesis (33). Other studies have shown that the insulin sensitivity of patients with MD is impaired, leading to increased blood sugar, and eventually leading to diabetes (34). Another study (35) that recruited 703 patients with MD showed that the prevalence of T2D in the MD was 21.2%, and it showed that being male, having hypertension, hypertriglyceridemia, BMI ≥30kg/m², and age ≥50 years old were significant risk factors for T2D in MD, and most of the risk factors for T2D were reversible. Antidepressant drugs (ADs), such as tricyclic antidepressants, mirtazapine, and sertraline, taken by depressed patients affect blood sugar and are associated with a higher risk of developing T2D. The pharmacological mechanism may be the higher degrees of occupancy on muscarinic receptors and H(1). Most studies published in the past two decades have not reported the positive effect of ADs on blood sugar control in diabetic patients (36, 37). In summary, we believe that both short-term depressive status and MD will increase the risk of T2D.

Although our results showed that there was no causal relationship between T2D and depression, T2D might have an impact on the progress of MD and depressive status. Several potential pathways between T2D and depression have been widely accepted, one of which is that the use of diabetes drugs will increase the incidence rate of depression, including the use of insulin, sulfonylurea drugs, and high-dose metformin (38). A study (25) found that T2D patients had a higher risk of developing MD, and as the number of risk factors within the recommended target range increased, the additional risk of developing MD decreased, which may be related to unhealthy lifestyles. Our study had both advantages and limitations. In order to avoid the influence of mixed risk factors, in the current study, we applied MR analysis. Due to its research design, it reveals causal relationships other than bias, which strengthens the causal inference of the association between depression and renal T2D risk. The study did not find a reverse causal relationship. Our research also has some limitations. Most of the samples in our research were from Europe. We still need further evidence to evaluate the relationship in other countries with different economic statuses and spanning additional ancestral backgrounds.

## Conclusion

Our study supports a causal relationship between MD and depressive status and the risk of developing T2D, while no causal relationship was found in the reverse direction. These findings highlight the importance of monitoring T2D risk in patients with depression, suggesting a need for regular screening and preventive measures in this population.

## Data Availability

Publicly available datasets were analyzed in this study. This data can be found here: https://www.finngen.fi/en; https://www.ukbiobank.ac.uk.
